# Ouabain Relieves Sleep Deprivation–Induced Anxiety‐Like Behavior in Mice by Suppressing Hippocampal Neuroinflammation and Oxidative Stress

**DOI:** 10.1002/brb3.71629

**Published:** 2026-07-24

**Authors:** Hanxiao Zhu, Wei Wang

**Affiliations:** ^1^ Department of Neurology, The First Affiliated Hospital of Dali University; Clinical Medical School, Health Science Center Dali University Dali China; ^2^ College of Basic Medical, Health Science Center Dali University Dali China

**Keywords:** anxiety‐like behavior, hippocampus, microglia, Na+/K+‐ATPase, neuroinflammation, ouabain, oxidative stress, sleep deprivation, Src/p38 MAPK/NF‐κB

## Abstract

**Background:**

Sleep insufficiency has become a global public health challenge and is closely associated with the onset of mood and anxiety disorders. Neuroinflammation and oxidative stress are considered key pathological substrates underlying these conditions. Ouabain is a prototypical cardiotonic glycoside and an endogenous ligand of Na^+^/K^+^‐ATPase. In recent years, ouabain has been reported to exert anti‐inflammatory and neuroprotective effects; however, whether it can ameliorate sleep deprivation (SD)‐associated affective abnormalities remains unclear.

**Methods:**

Using a 72‐h modified multiple‐platform SD model in male ICR mice, we investigated whether low‐dose ouabain administration (3 µg/kg, i.p.) alleviates anxiety‐like behaviors, as assessed by the open field test, and mitigates hippocampal inflammatory (cytokine TNF‐α, IL‐1β, IL‐4, and IL‐10) and redox disturbances (T‐AOC, SOD, GPx, MDA, and CAT), as measured by ELISA. In parallel, PLX5622 and pathway‐specific modulators were employed to explore the potential mechanisms underlying the beneficial effects of ouabain.

**Results:**

In this study, SD reduced center zone time in the open field by 56.05% without changing locomotor activity, increased hippocampal TNF‐α, IL‐1β, and MDA by 105.11%, 82.50%, and 89.82%, respectively, and decreased IL‐4, IL‐10, SOD, GPx, CAT, and T‐AOC by 54.28%, 47.22%, 44.96%, 51.95%, 52.14%, and 46.20%, respectively. Administration of low‐dose ouabain significantly reversed these changes. PLX5622‐mediated microglial depletion produced a partially similar protective profile, and pharmacological interference with Src/p38 MAPK/NF‐κB‐associated signaling attenuated the effect of ouabain.

**Conclusion:**

Collectively, these findings suggest that low‐dose ouabain mitigates acute SD‐induced anxiety‐like behavior, at least in part by suppressing hippocampal neuroinflammation and oxidative stress, and identify a potential signaling axis for further investigation.

## Graphical abstract

Low‐dose ouabain effectively counteracts acute SD‐induced oxidative stress in the hippocampus, restoring both enzymatic and non‐enzymatic antioxidant defenses and attenuating lipid peroxidation, thereby reversing acute SD‐induced anxiety‐like behavior. Moreover, ouabain's anxiolytic‐like effects appear to be mediated through the Src/p38 MAPK/NF‐κB signaling axis.

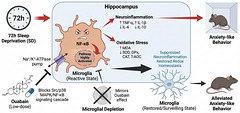

## 1 Introduction

In modern society, approximately one‐third of adults chronically sleep fewer than 7 h per night, and the prevalence of short sleep duration has not substantially declined over the past decade; similar trends have been observed across multiple countries and regions (Pankowska et al. 2023; Krause et al. 2017). Extensive animal studies and neuroimaging evidence indicate that both acute and chronic SD impair attention, working memory, and executive function, accompanied by reduced emotional regulation and enhanced negative affect. For example, in humans, one to several nights of sleep restriction can elicit anxiety and irritability. In rodents, short‐term SD commonly decreases exploration of the center zone in the open field and increases avoidance of potentially threatening environments in the elevated plus maze, while overall motor capacity often remains relatively preserved (Krause et al. 2017; W. Wang, Wang, et al. 2024; Zhang et al. 2025).

Physiologically, SD disrupts immune and endocrine homeostasis, activates central and peripheral monocyte–macrophage systems, promotes microglial and astrocytic activation, upregulates pro‐inflammatory mediators such as TNF‐α and IL‐1β, and engages inflammasome signaling, thereby increasing vulnerability to neuropsychiatric disorders (Besedovsky et al. 2019; Garbarino et al. 2021). In acute SD mice, microglial activation in the hippocampus leads to excessive synaptic engulfment and ultimately cognitive impairment (Li et al. 2023). In parallel, SD induces oxidative stress, characterized by enhanced lipid peroxidation, reduced total antioxidant capacity, decreased activities of antioxidant enzymes (e.g., superoxide dismutase and catalase), and mitochondrial dysfunction. When severe or prolonged, these changes may trigger ferroptosis and apoptosis, particularly in high metabolic‐demand regions such as the hippocampus and prefrontal cortex (W. Wang, Wang, et al. 2024; Neculicioiu et al. 2023). Recent work shows that acute SD induces marked region‐specific transcriptional responses, with particularly prominent alterations in the hippocampus (Lyons et al. 2020). Although SD‐induced anxiety and affective dysregulation are increasingly recognized as outcomes of multiple molecular pathways—including neuroinflammation, redox imbalance, aberrant limbic network activity, hyperactivation of the hypothalamic–pituitary–adrenal axis, and glutamatergic excitotoxicity (Lund et al. 2010; Zhang et al. 2025)—the critical molecular nodes that shape anxiety‐like behavior remain insufficiently defined, and actionable targets for intervention are limited.

Ouabain is a plant‐derived cardiotonic glycoside initially isolated from *Strophanthus gratus* and *Acokanthera schimperi*. Historically used as an arrow poison in parts of Africa, it was later introduced into modern medicine for its positive inotropic action in congestive heart failure (Elendu et al. 2025). Since the 1990s, endogenous substances highly similar or identical to plant ouabain have been detected in mammalian plasma and tissues, leading to the concept of “endogenous digitalis‐like factors,” proposed to participate in long‐term regulation of body fluid volume and blood pressure (Boulanger et al. 1993; Hamlyn and Blaustein 2016; Fender et al. 2024). At the molecular level, ouabain binds the Na^+^/K^+^‐ATPase α‐subunit with high affinity. At micromolar concentrations, it inhibits pump function and alters transmembrane Na^+^/K^+^ electrochemical gradients; at nanomolar or lower concentrations, it primarily acts as a ligand to engage Na^+^/K^+^‐ATPase‐associated signalosomes and activate pathways such as Src, ERK, and PI3K/Akt, affecting neurons, glial cells, and peripheral immune cells (Schoner and Scheiner‐Bobis 2007; Leite et al. 2015; Hamlyn and Blaustein 2016; Leite et al. 2022). Through these dual roles in ion transport and signal transduction, ouabain regulates vascular tone, myocardial contraction, and renal sodium handling, and also modulates synaptic transmission and cell survival in the central nervous system. Dysregulated endogenous ouabain levels have been linked to hypertension, pregnancy‐related disorders, kidney disease, and certain neurological conditions (Boulanger et al. 1993; Hamlyn and Blaustein 2016; Blaustein and Hamlyn 2020; Elendu et al. 2025).

Recent work suggests that low‐dose ouabain exerts robust anti‐inflammatory and immunomodulatory actions, including inhibition of NF‐κB and p38 MAPK signaling, regulation of neutrophil migration, and modulation of T/B lymphocyte responses. In the brain, ouabain has been reported to attenuate glutamate excitotoxicity, promote myelin basic protein synthesis, regulate glutamate transport, and bias microglia toward an anti‐inflammatory phenotype, thereby conferring neuroprotection in models of traumatic brain injury, Alzheimer's disease, and inflammatory neuropathy (Leite et al. 2015, 2022; C. Wang et al. 2018; Garcia et al. 2023; D. Wang, Liu, et al. 2024). Additionally, Ouabain has also been found to have potent antioxidant effects, further ameliorating neuropathological outcomes (Garcia et al. 2019). Nonetheless, studies addressing ouabain in SD‐related behavioral and pathophysiological alterations remain scarce, and whether ouabain modulates SD‐induced neuroinflammation and oxidative stress to improve anxiety‐like behavior has not been reported. Therefore, we hypothesized that low‐dose ouabain attenuates SD‐induced anxiety‐like behavior by limiting hippocampal neuroinflammation and oxidative stress, at least in part through modulation of Na^+^/K^+^‐ATPase‐linked Src/p38 MAPK/NF‐κB signaling. Based on this, using an acute SD mouse model, we administered ouabain and evaluated its effects on SD‐induced anxiety‐like behavior, hippocampal neuroinflammation, and oxidative stress.

## 2 Materials and Methods

### 2.1 Animals

Male ICR mice (7–8 weeks old, 32–43 g) were purchased from Beijing Vital River Laboratory Animal Technology Co., Ltd. Mice were housed in a SPF facility under controlled temperature (22 ± 2°C) and humidity (50%–60%) with a 12 h light/12 h dark cycle (lights on from 8:00 a.m. to 8:00 p.m.). Food and water were available ad libitum. All experimental procedures complied with the Guidelines for the Care and Use of Laboratory Animals issued by the Chinese National Research Council (2006) and were approved by the Animal Ethics Committee of The First Affiliated Hospital of Dali University (Approval No. DFY20250122001). A total of 140 mice were used across all experimental groups. Animals were housed at a density of five mice per cage before the SD procedure. Mice were acclimatized for 14 days after arrival. During SD, animals were monitored at 12 h intervals for general activity, grooming, posture, food/water access, signs of hypothermia or excessive stress. Every effort was made to minimize animal number and suffering; sample sizes (*N*) are specified in the figure legends.

### 2.2 SD Model

SD was performed using a modified multiple‐platform water tank method as previously described (W. Wang, Wang, et al. 2024). The SD apparatus consisted of an opaque plastic water tank containing 12 small circular platforms (∼3 cm diameter, ∼7 cm height) evenly spaced within the tank. Water level was adjusted to ∼3 cm below the platform tops, allowing mice to stand and rest while awake; during rapid eye movement sleep or deep non‐rapid eye movement sleep, reduced muscle tone causes mice to fall into water and awaken. Control mice were placed in an identical tank but with a large platform (∼12 cm diameter) permitting normal sleep. SD lasted 72 h and began at a fixed time during the light phase. Food and water were accessible via a mesh rack on top of the tank, and water temperature was maintained at ∼25 ± 1°C. Mice were acclimated for 14 days after arrival and were gently handled daily to reduce stress. Animals were randomly assigned to control or SD groups, and experimenters were blinded to group allocation during behavioral testing.

### 2.3 Open Field Test (OFT)

**FIGURE 1:**
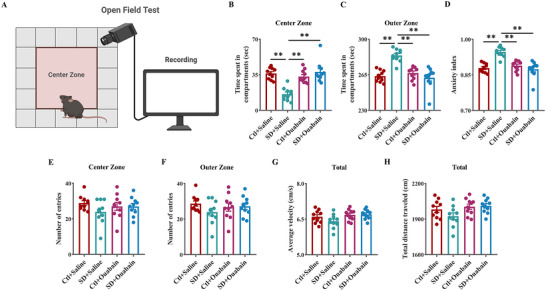
Ouabain alleviates acute sleep deprivation–induced anxiety‐like behavior. (A) Schematic of the open field test. (B) Time spent in the center zone. (C) Time spent in the outer zone. (D) Anxiety index (peripheral time/total time). (E) Number of center entries. (F) Number of peripheral entries. (G) Mean speed. (H) Total distance traveled. *N* = 10/group. Ctl + Ouabain, control group treated with ouabain; Ctl + Saline, control group treated with saline; SD + Ouabain, acute sleep deprivation group treated with ouabain; SD + Saline, acute sleep deprivation group treated with saline. *p < 0.05, **p < 0.01.

The OFT apparatus was an opaque plastic box (100 cm × 100 cm × 100 cm) with the floor divided into 25 equal squares (20 cm × 20 cm). The central zone was defined as the middle nine squares, and the remaining area was defined as the periphery (Figure 1A). Mice were habituated to the testing room for 30 min before testing, then gently placed in the center and allowed to explore freely for 5 min. Behavior was recorded and analyzed using an automated tracking system (XR‐XM101, Xinruan, Shanghai, China), yielding total distance traveled, time spent in the center, and number of center entries. The box was thoroughly cleaned with 75% ethanol and dried between trials to eliminate odor cues.

### 2.4 ELISA for Inflammatory Cytokines

Immediately after behavioral testing, mice were deeply anesthetized and rapidly decapitated. Bilateral hippocampi were dissected on ice, snap‐frozen in liquid nitrogen, and stored at −80°C. Levels of TNF‐α (JL10484; Jonlnbio, Shanghai, China), IL‐1β (JL18442; Jonlnbio), IL‐4 (JL20266; Jonlnbio), and IL‐10 (JL20242; Jonlnbio) were measured by ELISA according to the manufacturers’ protocols. Briefly, approximately 20 mg of hippocampal tissue was placed in 200 µL of prechilled RIPA lysis buffer (CW2333S, CWBIO, Beijing, China) and homogenized at 4°C using a tissue homogenizer at 60 Hz for two to three cycles, with each cycle lasting 3 min, to ensure complete lysis. The lysates were then centrifuged at 12,000 × *g* for 8 min at 4°C using a refrigerated centrifuge. The supernatants were carefully transferred to fresh 1.5‐mL microcentrifuge tubes. Protein concentrations were determined using a BCA protein assay kit (CW0014, CWBIO, Beijing, China), and absorbance was measured at 570 nm with a microplate reader. Standards and samples were added to pre‐coated 96‐well plates, followed by incubation with biotinylated antibody and streptavidin‐HRP. After TMB development and reaction termination, absorbance was read at 450 nm. Cytokine concentrations were calculated from standard curves and normalized to total protein measured by BCA assay. Each sample was measured in at least duplicate wells, and values were averaged before statistical analysis.

### 2.5 Measurement of Oxidative Stress

Parallel hippocampal samples were homogenized for oxidative stress assays. Commercial kits were used to quantify total antioxidant capacity (T‐AOC; S0116; Beyotime), superoxide dismutase activity (SOD; S0101S; Beyotime, Shanghai, China), glutathione peroxidase activity (GPx; S0056; Beyotime), glutathione reductase activity (GR; S0055; Beyotime), catalase activity (CAT; S0051; Beyotime), and malondialdehyde content (MDA; S0131S; Beyotime). Procedures followed manufacturers’ instructions. Optical density was measured using a multimode microplate reader (Varioskan Lux, Thermo Fisher, USA). All results were normalized to protein concentration, and each sample was assayed in at least duplicate wells.

### 2.6 Drug Administration

Ouabain (≥ 98% purity; CAS 11018‐89‐6; Sigma, St. Louis, USA), SU6656 (HY‐B0789; MedChemExpress, USA), anisomycin (HY‐18982; MedChemExpress), and TNF‐α (HY‐P1860; MedChemExpress) were dissolved in DMSO and then diluted with normal saline to appropriate concentrations before use. Mice in the ouabain group received the first dose (3 µg/kg) 24 h before the start of SD, followed by once‐daily administration until completion of 72 h SD (four injections total). Control mice received equivalent volumes of saline containing DMSO. For inhibitor/agonist interventions, SU6656 (30 mg/kg, i.p.), anisomycin (60 mg/kg, i.p.), or TNF‐α (3 µg/kg, i.p.) was administered 30 min before each ouabain injection.

### 2.7 PLX5622 Treatment

PLX5622 chow (HY‐114153C; MedChemExpress) was prepared by adding PLX5622 to AIN‐76A base diet at 1200 mg/kg (1200 ppm). Mice received 1200 mg/kg/day for the first 8 days, followed by 300 mg/kg/day for the next 10 days. Control mice received the same base diet (AIN‐76A) without PLX5622. Animals were randomly assigned to CON, SD, and PLX5622 groups. PLX5622 feeding began 14 days prior to SD and continued until sacrifice.

### 2.8 Statistical Analysis

Data are presented as mean ± SEM. Normality was assessed by the Shapiro–Wilk test, and variance homogeneity was examined using the Brown–Forsythe test. Two group comparisons were performed using two‐tailed unpaired Student's *t*‐test. Multiple group comparisons were analyzed by one‐way ANOVA followed by Tukey's post hoc test. Statistical analyses were conducted in GraphPad Prism 9 (GraphPad Software, Inc.). A value of *p* < 0.05 was considered statistically significant (*p < 0.05; **p < 0.01).

## 3 Results

### 3.1 Ouabain Alleviates Acute SD‐Induced Anxiety‐Like Behavior

After 72 h of SD, ICR mice displayed a robust anxiety‐like phenotype in the OFT. Compared with control mice, SD reduced the time spent in the center zone by 56.05% (Figure 1B; *p* < 0.01) and increased the time spent in the periphery by 7.72% (Figure 1C; *p* < 0.01), resulting in a 7.73% increase in the anxiety‐like index (Figure 1D; *p* < 0.01). In contrast, the number of entries into each zone (Figure 1E,F; *p* > 0.05), mean speed (Figure 1G; *p* > 0.05), and total distance traveled (Figure 1H; *p* > 0.05) were not significantly altered, indicating that SD primarily affected emotion‐related behavior rather than general locomotion. Systemic administration of low‐dose ouabain had little effect on behavior in non‐deprived mice but substantially attenuated SD‐induced anxiety‐like behavior: compared with SD + Saline animals, SD + Ouabain mice spent 137.01% more time in the center zone (Figure 1B; *p* < 0.01), 7.70% less time in the periphery (Figure 1C; *p* < 0.01), and showed a 7.69% reduction in the anxiety‐like index (Figure 1D; *p* < 0.01). Again, ouabain did not significantly alter zone entries, mean speed, or total distance traveled (Figure 1E–H).

These data suggest that low‐dose ouabain reverses SD‐induced anxiety‐like behavior without producing sedation or suppressing spontaneous locomotion.

### 3.2 Ouabain Suppresses SD‐Induced Hippocampal Neuroinflammation and Oxidative Stress

**FIGURE 2:**
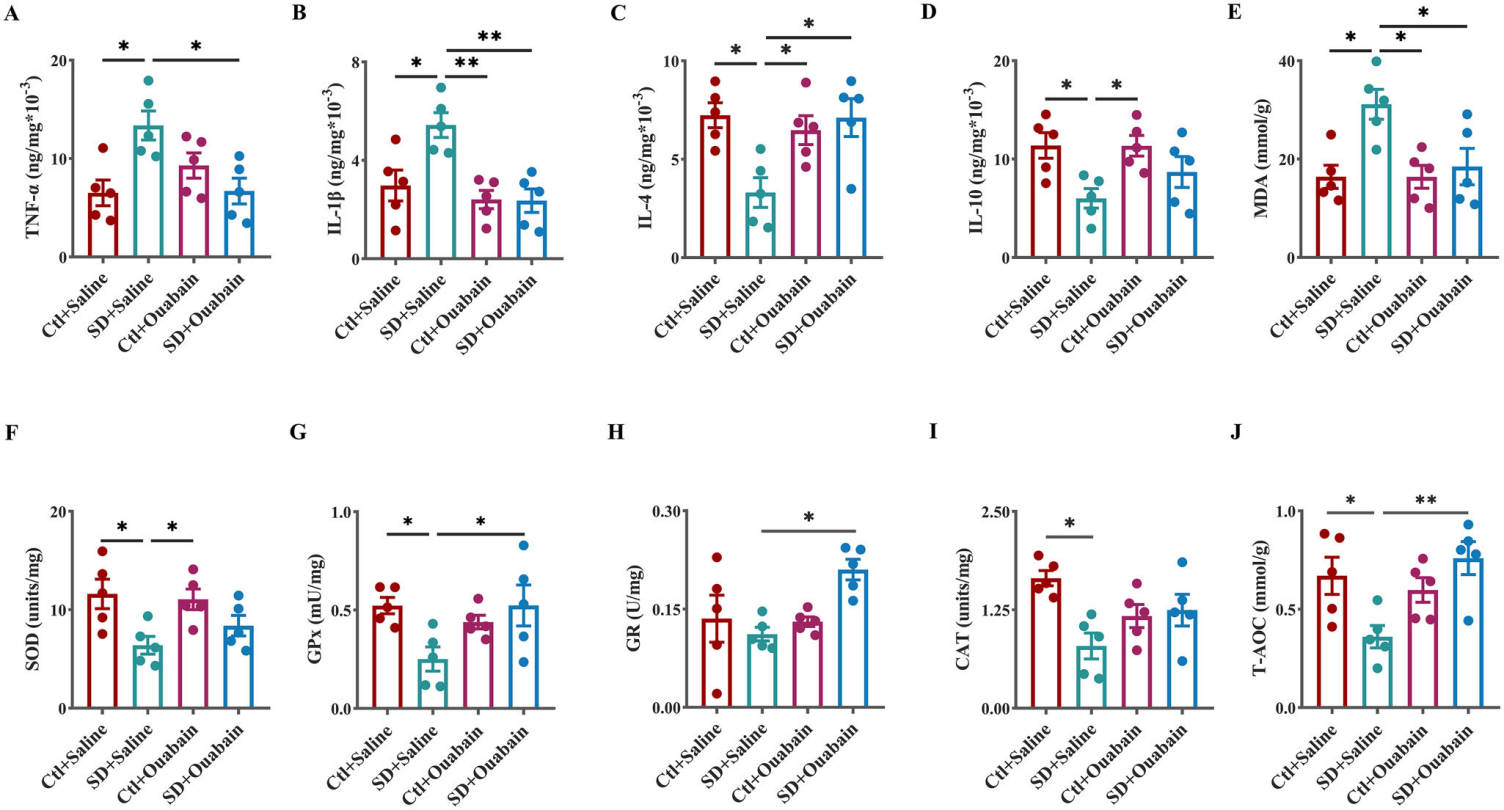
Ouabain attenuates hippocampal inflammatory responses and oxidative stress induced by acute sleep deprivation. Hippocampal levels of TNF‐α (A), IL‐1β (B), IL‐4 (C), and IL‐10 (D). ELISA detection of MDA (E), SOD (F), GPx (G), GR (H), CAT (I), and T‐AOC (J). *N* = 5/group. Ctl + Ouabain, control group treated with ouabain; Ctl + Saline, control group treated with saline; SD + Ouabain, acute sleep deprivation group treated with ouabain; SD + Saline, acute sleep deprivation group treated with saline. *p < 0.05, **p < 0.01.

After 72 h SD, hippocampal pro‐inflammatory cytokine TNF‐α (Figure 2A; *p* < 0.05) and IL‐1β (Figure 2B; *p* < 0.05) levels were significantly elevated by 105.11% and 82.50%, respectively, relative to controls, whereas anti‐inflammatory cytokines IL‐4 (Figure 2C; *p* < 0.05) and IL‐10 (Figure 2D; *p* < 0.05) were reduced by 54.28% and 47.22%, respectively, indicating a shift toward a pro‐inflammatory state. Ouabain treatment significantly mitigated these alterations (Figure 2A–D). Compared with SD + Saline mice, SD + Ouabain mice exhibited 49.88% lower TNF‐α (Figure 2A; *p* < 0.05) and 56.55% lower IL‐1β levels (Figure 2B; *p* < 0.01), together with 115.02% and 44.48% increases in IL‐4 (Figure 2C; *p* < 0.05) and IL‐10 (Figure 2D; *p* > 0.05), respectively. Together, these findings suggest that ouabain suppresses SD‐induced hippocampal neuroinflammation and promotes a more anti‐inflammatory cytokine.

We next examined oxidative stress markers in the hippocampus. Acute SD increased MDA levels by 89.82% (Figure 2E; *p* < 0.05), consistent with enhanced lipid peroxidation, and reduced SOD (Figure 2F; *p* < 0.05), GPx (Figure 2G; *p* < 0.05), CAT (Figure 2I; *p* < 0.05), and T‐AOC (Figure 2J; *p* < 0.05) by 44.96%, 51.95%, 52.14%, and 46.20%, respectively (Figure 2F–J), confirming that SD imposes substantial oxidative burden on hippocampal tissue. In addition, ouabain markedly improved these oxidative parameters in SD mice (Figure 2E–J). Relative to SD + Saline animals, SD + Ouabain mice showed a 40.66% reduction in MDA (Figure 2E; *p* < 0.05), together with 31.22%, 108.35%, 57.64%, and 110.82% increases in SOD (Figure 2F; *p* > 0.05), GPx (Figure 2G; *p* < 0.05), CAT (Figure 2I; *p* > 0.05), and T‐AOC (Figure 2J; *p* < 0.01), respectively. Ouabain alone had minimal impact on oxidative parameters in control mice. These results indicate that ouabain effectively counteracts SD‐induced oxidative stress in the hippocampus, restoring both enzymatic and nonenzymatic antioxidant defenses and reducing lipid peroxidation.

### 3.3 Microglial Depletion Mitigates SD‐Induced Anxiety‐Like Behavior and Restores Inflammatory/Redox Balance

**FIGURE 3:**
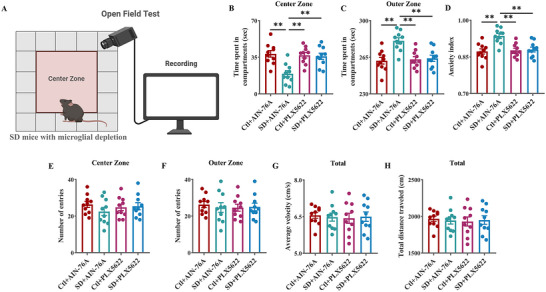
Microglial depletion improves acute sleep deprivation–induced anxiety‐like behavior. (A) Schematic of the open field test. (B) Time spent in the center zone. (C) Time spent in the outer zone. (D) Anxiety index (peripheral time/total time). (E) Number of center entries. (F) Number of peripheral entries. (G) Mean speed. (H) Total distance traveled. *N* = 10/group. Ctl + AIN‐76A, control group treated with base diet; Ctl + PLX5622, control group treated with PLX5622; SD + AIN‐76A, acute sleep deprivation group treated with base diet; SD + PLX5622, acute sleep deprivation group treated with PLX5622. *p < 0.05, **p < 0.01.

To test whether microglia‐mediated neuroinflammation contributes to SD‐induced anxiety‐like behavior, microglia were depleted with PLX5622. Under baseline conditions, PLX5622 treatment did not induce overt anxiety‐like behavior or locomotor abnormalities (Figure 3A–H). However, in the context of SD, PLX5622 significantly alleviated anxiety‐like behavior. Compared with the corresponding SD group without PLX5622, microglial depletion increased center time by 86.56% (Figure 3B; *p* < 0.01), decreased peripheral time by 59.42% (Figure 3C; *p* < 0.01), and reduced the anxiety‐like index by 59.41% (Figure 3D; *p* < 0.01).

**FIGURE 4:**
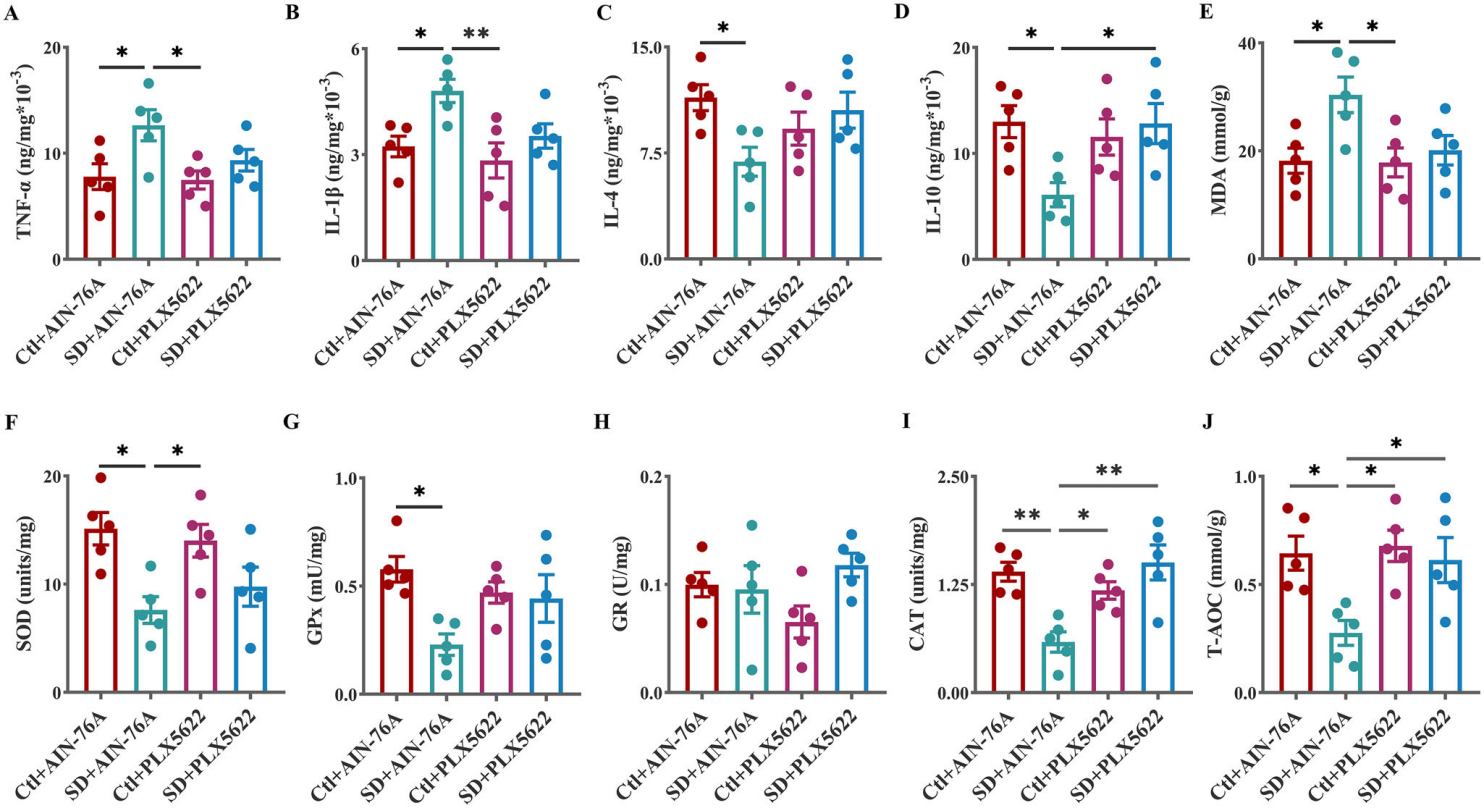
Microglial depletion alleviates acute sleep deprivation–induced hippocampal inflammation and oxidative stress. Hippocampal levels of TNF‐α (A), IL‐1β (B), IL‐4 (C), IL‐10 (D). ELISA detection of MDA (E), SOD (F), GPx (G), GR (H), CAT (I), and T‐AOC (J). *N* = 5/group. Ctl + AIN‐76A, control group treated with base diet; Ctl + PLX5622, control group treated with PLX5622; SD + AIN‐76A, acute sleep deprivation group treated with base diet; SD + PLX5622, acute sleep deprivation group treated with PLX5622. *p < 0.05, **p < 0.01.

Furthermore, microglial depletion also attenuated SD‐induced elevations of pro‐inflammatory cytokines (Figure 4A,B). Relative to the corresponding SD group, PLX5622 reduced hippocampal TNF‐α (Figure 4A; *p* > 0.05) and IL‐1β (Figure 4B; *p* > 0.05) by 26.12% and 26.52%, respectively, while increasing IL‐4 (Figure 4C; *p* > 0.05) and IL‐10 (Figure 4D; *p* < 0.05) by 53.07% and 110.13%. PLX5622 also lowered MDA by 33.79% (Figure 4E; *p* > 0.05) and increased SOD (Figure 4F; *p* > 0.05), GPx (Figure 4G; *p* > 0.05), CAT (Figure 4I; *p* < 0.01), and T‐AOC (Figure 4J; *p* < 0.05) by 28.34%, 93.02%, 157.08%, and 123.59%, respectively.

These results support a critical role of microglia in coupling SD to hippocampal inflammation/redox dysregulation and anxiety‐like behavior.

### 3.4 The Src/p38 MAPK/NF‐κB Pathway Mediates the Anxiolytic‐Like Effect of Ouabain

**FIGURE 5:**
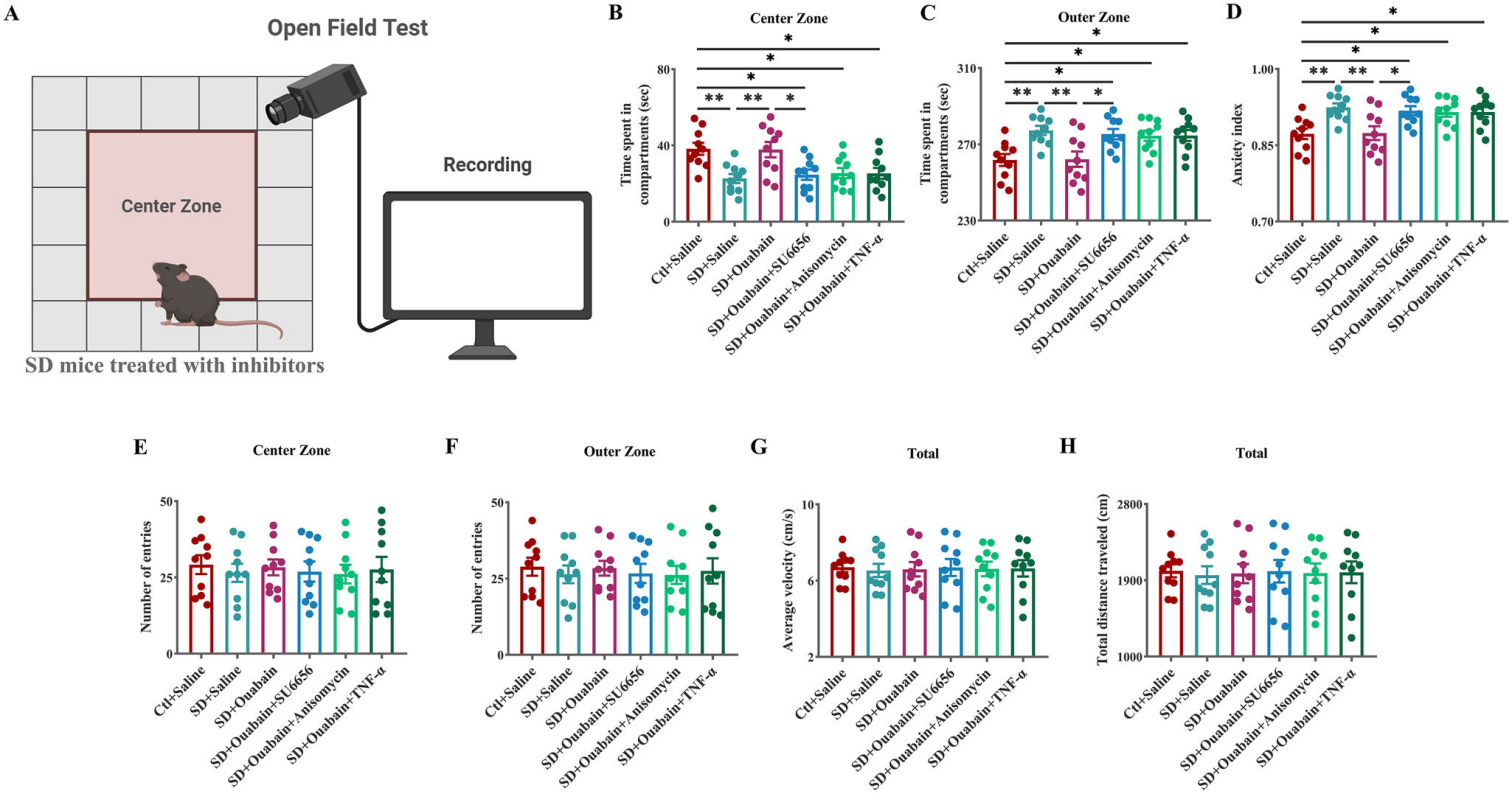
Ouabain's anxiolytic‐like effect depends on the Src/p38 MAPK/NF‐κB pathway. (A) Schematic of the open field test. (B) Time spent in the center zone. (C) Time spent in the outer zone. (D) Anxiety index (peripheral time/total time). (E) Number of center entries. (F) Number of peripheral entries. (G) Mean speed. (H) Total distance traveled. *N* = 10/group. Ctl, control group; SD, acute sleep deprivation group. *p < 0.05, **p < 0.01.

**FIGURE 6:**
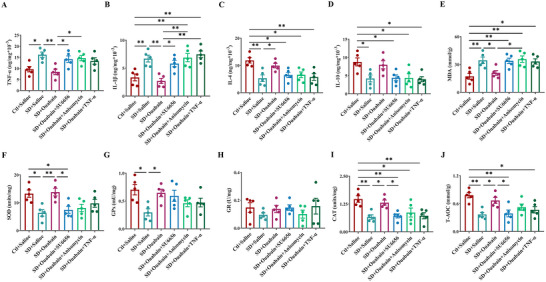
Ouabain's antioxidant and anti‐inflammatory effects depends on the Src/p38 MAPK/NF‐κB pathway. Hippocampal levels of TNF‐α (A), IL‐1β (B), IL‐4 (C), IL‐10 (D). ELISA detection of MDA (E), SOD (F), GPx (G), GR (H), CAT (I), and T‐AOC (J). *N* = 5/group. Ctl, control group; SD, acute sleep deprivation group. *p < 0.05, **p < 0.01.

Previous studies suggest that ouabain modulates immune signaling by inhibiting p38 MAPK and NF‐κB activation and nuclear translocation, thereby regulating cytokine expression and exerting neuroprotective effects (Schoner and Scheiner‐Bobis 2007; Kinoshita et al. 2014; Leite et al. 2015, 2022). To examine the signaling mechanisms under SD, we employed pharmacological interventions targeting downstream pathways following ouabain binding to Na^+^/K^+^‐ATPase. The Src family kinase inhibitor SU6656, the p38 MAPK activator anisomycin, and the NF‐κB pathway activator TNF‐α each attenuated the behavioral benefits of ouabain (Figure 5A–H). Specifically, center time was reduced by 35.23% (Figure 5B; *p* < 0.05), 32.59% (Figure 5B; *p* = 0.0585), and 32.96% (Figure 5B; *p* = 0.0540), respectively, peripheral time was increased by 5.02% (Figure 5C; *p* < 0.05), 4.70% (Figure 5C; *p* = 0.0585), and 4.76% (Figure 5C; *p* = 0.0540), respectively, and the anxiety‐like index was increased by 5.02% (Figure 5D; *p* < 0.05), 4.70% (Figure 5D; *p* = 0.0585), and 4.76% (Figure 5D; *p* = 0.0540), respectively. Moreover, these interventions blunted the anti‐inflammatory (Figure 6A–D) and antioxidant (Figure 6E–J) effects of ouabain in SD mice.

Collectively, these data indicate that ouabain's anxiolytic‐like effects in SD may depend on the Src/p38 MAPK/NF‐κB signaling axis.

## 4 Discussion

Sleep insufficiency is highly prevalent worldwide, with nearly one‐third of adults sleeping less than 7 h per night. This pattern not only increases cardiometabolic risk but also substantially elevates the incidence of affective disorders, particularly anxiety and depression (Pankowska et al. 2023; Krause et al. 2017; Ramos et al. 2023). Extensive human and animal research has established that SD impairs attention, memory, and executive function and increases emotional reactivity and anxiety (Walker and van der Helm 2009; Krause et al. 2017; W. Wang, Wang, et al. 2024; Zhang et al. 2025; Zhu et al. 2026). Here, acute 72 h SD decreased exploration of the open field center and increased anxiety‐like behavior without altering total locomotor distance, indicating that under our model and time scale, SD primarily induced affective changes rather than nonspecific motor deficits. This pattern aligns with prior findings in humans and rodents showing that even brief SD can increase anxiety and negative affect, often accompanied by heightened amygdala activity and weakened prefrontal regulation (Shi et al. 2023; W. Wang, Wang, et al. 2024; Zhang et al. 2025). Although the OFT revealed a consistent reduction in center exploration without locomotor suppression, we acknowledge that the use of a single principal anxiety‐related paradigm limits behavioral breadth. Future studies should incorporate additional tests such as the elevated plus maze and light‐dark box.

Notably, some animal studies have reported no anxiogenic effect or even anxiolytic‐like outcomes following SD, or have linked SD to manic‐like behavior (Martinez‐Gonzalez et al. 2004; Young et al. 2011; Shi et al. 2023). Such discrepancies may reflect differences in baseline affective state (e.g., severe depression vs. healthy animals), SD paradigms (partial nocturnal SD with phase advance vs. sustained near‐complete SD), SD duration, and recovery strategies. Moreover, SD may engage distinct neural circuits across brain regions and neurotransmitter systems: short‐term enhancement of monoaminergic transmission may confer rapid antidepressant‐like effects, whereas hippocampal and amygdala inflammation/oxidative stress may drive anxiety‐like behavior.

Accumulating evidence indicates that sleep loss is a potent trigger of neuroinflammation and oxidative stress, observed in biofluid assays from short‐term SD in healthy individuals and in brain tissue analyses across multiple SD animal models (Hurtado‐Alvarado et al. 2013; Krause et al. 2017; Besedovsky et al. 2019; Garbarino et al. 2021; Neculicioiu et al. 2023; W. Wang, Wang, et al. 2024; Zhang et al. 2025). In the present study, acute SD increased hippocampal TNF‐α and IL‐1β while decreasing IL‐4 and IL‐10, accompanied by increased MDA and decreased T‐AOC and antioxidant enzyme activities, collectively indicating a transition from homeostasis toward a pro‐inflammatory, pro‐oxidant state. Importantly, neuroinflammation and oxidative stress can mutually amplify one another: pro‐inflammatory cytokines impair mitochondrial function and suppress antioxidant defenses, whereas reactive oxygen species and lipid peroxidation activate NF‐κB and inflammasomes, forming a feed‐forward loop (Teleanu et al. 2022; Neculicioiu et al. 2023). Consistent with this concept, microglial depletion with PLX5622 improved anxiety‐like behavior and reduced both inflammatory and oxidative perturbations after SD. These findings parallel observations in other disease models, where CSF1R inhibition alleviates depression‐/anxiety‐like behavior and cognitive deficits while reducing neuroinflammatory markers (Hatton and Duncan 2019; Bhatia et al. 2023; Kokkosis et al. 2024). Together, our results support a model in which SD activates microglia to drive hippocampal neuroinflammation and oxidative stress, thereby impairing circuit function and promoting anxiety‐like behavior.

Traditionally, ouabain has been viewed primarily as a cardiotonic glycoside that enhances myocardial contractility by inhibiting cardiac Na^+^/K^+^‐ATPase (Elendu et al. 2025). However, emerging evidence indicates that low‐dose ouabain exerts broad anti‐inflammatory and antioxidant actions, including inhibition of NF‐κB and p38 MAPK signaling and promotion of anti‐inflammatory microglial polarization, leading to neuroprotection in models of traumatic brain injury, Alzheimer's disease, and LPS‐induced neuroinflammation (Leite et al. 2015, 2022; C. Wang et al. 2018; Garcia et al. 2019, 2023; D. Wang, Liu, et al. 2024). In our SD model, low‐dose ouabain improved anxiety‐like behavior without affecting locomotion and concomitantly normalized pro‐/anti‐inflammatory mediators and restored redox homeostasis, consistent with dual immunomodulatory and antioxidant effects. Interestingly, SD is increasingly recognized as a neuroimmune‐redox challenge rather than a purely behavioral perturbation. In the hippocampus, Wang et al. reported that melatonin attenuates SD‐induced anxiety‐like behavior by reducing oxidative stress, NF‐κB‐related neuroinflammation, autophagy, and apoptosis (X. Wang et al. 2021), and Kang et al. further showed that Hsp70 ameliorates SD‐induced anxiety‐like behavior and cognitive impairment while restoring pCREB/BDNF signaling and reducing microglia‐associated neuroinflammation (Kang et al. 2023). Together with recent findings, these studies support the view that hippocampal inflammatory activation and oxidative stress form a mutually amplifying pathogenic loop that contributes to sleep loss‐related affective dysfunction (Lutfy et al. 2025; Alam‐ElDein, Faraag, et al. 2026; Alam‐ElDein, Shaker, et al. 2026). Mechanistically, given the bidirectional reinforcement between oxidative stress and neuroinflammation (Teleanu et al. 2022; Neculicioiu et al. 2023), ouabain may act by modulating Na^+^/K^+^‐ATPase–linked signaling and ionic homeostasis to improve intracellular redox balance, thereby restraining microglial activation and inflammatory mediator release; direct immune signaling effects may also contribute. Using pathway‐directed pharmacology, we found that the beneficial effects of ouabain may depended on the Src/p38 MAPK/NF‐κB cascade, supporting a signaling‐based mechanism downstream of Na^+^/K^+^‐ATPase. Notably, we did not assess BDNF or GFAP in the present study. As a result, although our data support anti‐inflammatory and antioxidant effects of ouabain, they do not yet directly establish whether these changes are accompanied by restoration of hippocampal neuroplasticity or attenuation of astroglial reactivity. Unlike previous studies that examined ouabain primarily in peripheral/LPS‐driven inflammatory paradigms or in sleep‐wake regulation, our work tests whether low‐dose ouabain can rescue SD‐induced anxiety‐like behavior while linking behavioral protection to hippocampal inflammatory/redox normalization, microglial depletion, and pathway‐directed pharmacology.

In our study, male mice were used in this initial mechanistic study to minimize one layer of endocrine variability related to estrous cycling. However, this design limits generalizability, and future studies should include both sexes to determine whether the effects of SD and ouabain are sex‐dependent. Meanwhile, the model used here represents acute 72 h SD and therefore may not fully capture the neurobiological complexity of chronic or recurrent sleep loss in clinical settings. Moreover, our findings suggest that the protective effect of ouabain may involve a Na^+^/K^+^‐ATPase‐linked Src/p38 MAPK/NF‐κB signaling axis. Yet, direct protein‐level validation will be required to establish this mechanism more definitively. Although the hippocampus was the focus of the present study, acute SD affects multiple brain regions, and region‐specific contributions from structures such as prefrontal cortex, neocortex, hypothalamus, and thalamus warrant future investigation. Finally, ouabain is a cardiotonic glycoside/ Na^+^/K^+^‐ATPase ligand with well‐established cardiac pharmacology, and cardiac glycosides are known to have a relatively narrow therapeutic range. Therefore, even though the dose used here was low, our data should not be interpreted as demonstrating translational safety. ECG monitoring, serum chemistry/electrolytes, body‐weight trajectory, and histopathological safety assessment were not performed and will be necessary in future work.

## 5 Conclusion

In conclusion, this study provides multi‐level evidence—behavioral, inflammatory, and redox—that low‐dose ouabain alleviates acute SD‐induced anxiety‐like behavior, at least in part by suppressing microglia‐associated hippocampal neuroinflammation and oxidative stress. These findings offer a mechanistic rationale for repurposing cardiotonic glycosides or targeting the ouabain–Na^+^/K^+^‐ATPase signaling axis to counteract sleep loss related affective disturbances, and further underscore the fundamental importance of adequate, high‐quality sleep for neuroimmune–redox homeostasis and emotional well‐being. Further studies using both sexes, different durations and chronic SD models, additional anxiety assays, direct pathway validation, neuroplasticity/glial markers, and safety profiling are required.

## Author Contributions

**Hanxiao Zhu**: data curation, formal analysis, writing – original draft, funding acquisition. **Wei Wang**: conceptualization, data curation, formal analysis, funding acquisition, supervision.

## Funding

This research was funded by a grant from the Youth Project of the Joint Project of Basic Research of Local Universities in Yunnan Province (Grant No. 202401BA070001‐010) and a grant from the Doctoral Research Initiation Fund Project of Dali University (Grant No. KYBS2026023).

## Ethics Statement

All experimental procedures complied with the Guidelines for the Care and Use of Laboratory Animals issued by the Chinese National Research Council (2006) and were approved by the Animal Ethics Committee of The First Affiliated Hospital of Dali University (Approval No. DFY20250122001).

## Consent

The authors have nothing to report.

## Conflicts of Interest

The authors declare no conflicts of interest.

## Data Availability

The data that support the findings of this study are available on request from the corresponding author.

## References

[brb371629-bib-0001] Alam‐ElDein, K. M. , A. H. I. Faraag , N. A. El‐Yamany , et al. 2026. “Studying the Potential Ameliorative Effect of Biosynthesized Selenium Nanoparticles Using Epigallocatechin Gallate against Depression in Rats.” Frontiers in Pharmacology 16: 1691567. 10.3389/fphar.2025.1691567. 41608023 PMC12835393

[brb371629-bib-0002] Alam‐ElDein, K. M. , A. A. S. Shaker , M. F. El‐Khadragy , et al. 2026. “Neuroprotective Effects of Rutin, Sodium Selenite, and Rutin‐Conjugated Selenium Nanoparticles in a Social Isolation Model.” Frontiers in Pharmacology 17: 1782734. 10.3389/fphar.2026.1782734. 42064794 PMC13125023

[brb371629-bib-0003] Besedovsky, L. , T. Lange , and M. Haack . 2019. “The Sleep‐Immune Crosstalk in Health and Disease.” Physiological Reviews 99, no. 3: 1325–1380. 10.1152/physrev.00010.2018. 30920354 PMC6689741

[brb371629-bib-0004] Bhatia, T. N. , A. S. Jamenis , M. Abbas , et al. 2023. “A 14‐Day Pulse of PLX5622 Modifies α‐Synucleinopathy in Preformed Fibril‐Infused Aged Mice of Both Sexes.” Neurobiology of Disease 184: 106196. 10.1016/j.nbd.2023.106196. 37315905 PMC10528721

[brb371629-bib-0005] Blaustein, M. P. , and J. M. Hamlyn . 2020. “Ouabain, Endogenous Ouabain and Ouabain‐Like Factors: The Na^+^ Pump/Ouabain Receptor, Its Linkage to NCX, and Its Myriad Functions.” Cell Calcium 86: 102159. 10.1016/j.ceca.2020.102159. 31986323

[brb371629-bib-0006] Boulanger, B. R. , M. P. Lilly , J. M. Hamlyn , J. Laredo , D. Shurtleff , and D. S. Gann . 1993. “Ouabain Is Secreted by the Adrenal Gland in Awake Dogs.” American Journal of Physiology 264, no. 3 Pt 1: E413–E419. 8460688 10.1152/ajpendo.1993.264.3.E413

[brb371629-bib-0007] Elendu, C. , D. C. Amaechi , T. C. Elendu , et al. 2025. “The Pump, the Exchanger, and the Holy Spirit: Tracing the 40‐Year Evolution of the Ouabain‐Na^+^ Pump Endocrine System Concept.” Annals of Medicine & Surgery 87, no. 7: 4281–4302. 10.1097/MS9.0000000000003438. 40851957 PMC12369745

[brb371629-bib-0008] Fender, J. , J. Klöcker , V. Boivin‐Jahns , U. Ravens , R. Jahns , and K. Lorenz . 2024. ““Cardiac Glycosides”—Quo Vaditis?—Past, Present, and Future?” Naunyn‐Schmiedeberg's Archives of Pharmacology 397, no. 12: 9521–9531. 10.1007/s00210-024-03285-3. 39007928 PMC11582269

[brb371629-bib-0009] Garbarino, S. , P. Lanteri , N. L. Bragazzi , N. Magnavita , and E. Scoditti . 2021. “Role of Sleep Deprivation in Immune‐Related Disease Risk and Outcomes.” Communications Biology 4, no. 1: 1304. 10.1038/s42003-021-02825-4. 34795404 PMC8602722

[brb371629-bib-0010] Garcia, I. J. P. , P. F. Kinoshita , L. N. D. E. Silva , et al. 2019. “Ouabain Attenuates Oxidative Stress and Modulates Lipid Composition in Hippocampus of Rats in Lipopolysaccharide‐Induced Hypocampal Neuroinflammation in Rats.” Journal of Cellular Biochemistry 120, no. 3: 4081–4091. 10.1002/jcb.27693. 30260008

[brb371629-bib-0011] Garcia, I. J. P. , P. F. Kinoshita , J. M. D. M. Valadares , et al. 2023. “Effect of Ouabain on Glutamate Transport in the Hippocampus of Rats With LPS‐Induced Neuroinflammation.” Biomedicines 11, no. 3: 920. 10.3390/biomedicines11030920. 36979899 PMC10045517

[brb371629-bib-0012] Hamlyn, J. M. , and M. P. Blaustein . 2016. “Endogenous Ouabain: Recent Advances and Controversies.” Hypertension 68, no. 3: 526–532. 10.1161/HYPERTENSIONAHA.116.06599. 27456525 PMC4982830

[brb371629-bib-0013] Hatton, C. F. , and C. J. A. Duncan . 2019. “Microglia Are Essential to Protective Antiviral Immunity: Lessons From Mouse Models of Viral Encephalitis.” Frontiers in Immunology 10: 2656. 10.3389/fimmu.2019.02656. 31798586 PMC6863772

[brb371629-bib-0014] Hurtado‐Alvarado, G. , L. Pavón , S. A. Castillo‐García , et al. 2013. “Sleep Loss as a Factor to Induce Cellular and Molecular Inflammatory Variations.” Clinical and Developmental Immunology 2013: 1–14. 10.1155/2013/801341. PMC3866883 24367384

[brb371629-bib-0015] Kang, Z. , Y. Lin , C. Su , S. Li , W. Xie , and X. Wu . 2023. “Hsp70 Ameliorates Sleep Deprivation‐Induced Anxiety‐Like Behavior and Cognitive Impairment in Mice.” Brain Research Bulletin 204: 110791. 10.1016/j.brainresbull.2023.110791. 37858682

[brb371629-bib-0016] Kinoshita, P. F. , L. M. Yshii , A. R. Vasconcelos , et al. 2014. “Signaling Function of Na,K‐ATPase Induced by Ouabain against LPS as an Inflammation Model in Hippocampus.” Journal of Neuroinflammation 11: 218. 10.1186/s12974-014-0218-z. 25551197 PMC4307894

[brb371629-bib-0017] Kokkosis, A. G. , M. M. Madeira , Z. Hage , et al. 2024. “Chronic Psychosocial Stress Triggers Microglial‐/Macrophage‐Induced Inflammatory Responses Leading to Neuronal Dysfunction and Depressive‐Related Behavior.” Glia 72, no. 1: 111–132. 10.1002/glia.24464. 37675659 PMC10842267

[brb371629-bib-0018] Krause, A. J. , E. B. Simon , B. A. Mander , et al. 2017. “The Sleep‐Deprived Human Brain.” Nature Reviews Neuroscience 18, no. 7: 404–418. 10.1038/nrn.2017.55. 28515433 PMC6143346

[brb371629-bib-0019] Leite, J. A. , A. K. D. A. Alves , J. G. M. Galvão , et al. 2015. “Ouabain Modulates Zymosan‐Induced Peritonitis in Mice.” Mediators of Inflammation 2015: 265798. 10.1155/2015/265798. 26078492 PMC4442290

[brb371629-bib-0020] Leite, J. A. , L. H. A. Cavalcante‐Silva , M. R. Ribeiro , G. de Morais Lima , C. Scavone , and S. Rodrigues‐Mascarenhas . 2022. “Neuroinflammation and Neutrophils: Modulation by Ouabain.” Frontiers in Pharmacology 13: 824907. 10.3389/fphar.2022.824907. 35173621 PMC8841582

[brb371629-bib-0021] Li, N. , S. Tan , Y. Wang , et al. 2023. “Akkermansia Muciniphila Supplementation Prevents Cognitive Impairment in Sleep‐Deprived Mice by Modulating Microglial Engulfment of Synapses.” Gut Microbes 15, no. 2: 2252764. 10.1080/19490976.2023.2252764. 37671803 PMC10484034

[brb371629-bib-0022] Lund, H. G. , B. D. Reider , A. B. Whiting , and J. R. Prichard . 2010. “Sleep Patterns and Predictors of Disturbed Sleep in a Large Population of College Students.” Journal of Adolescent Health 46, no. 2: 124–132. 10.1016/j.jadohealth.2009.06.016. 20113918

[brb371629-bib-0023] Lutfy, R. H. , A. M. Ashour , A. Khames , et al. 2025. “Targeting Oxidative Stress and Neuroinflammation: Epigallocatechin‐3‐Gallate‐Selenium Nanoparticles Mitigate Sleep Deprivation‐Induced Cortical Impairment.” International Journal of Molecular Sciences 26, no. 22: 11173. 10.3390/ijms262211173. 41303666 PMC12653894

[brb371629-bib-0024] Lyons, L. C. , S. Chatterjee , Y. Vanrobaeys , M. E. Gaine , and T. Abel . 2020. “Translational Changes Induced by Acute Sleep Deprivation Uncovered by TRAP‐Seq.” Molecular Brain 13, no. 1: 165. 10.1186/s13041-020-00702-5. 33272296 PMC7713217

[brb371629-bib-0025] Martinez‐Gonzalez, D. , W. Obermeyer , J. L. Fahy , M. Riboh , N. H. Kalin , and R. M. Benca . 2004. “REM Sleep Deprivation Induces Changes in Coping Responses That Are Not Reversed by Amphetamine.” Sleep 27, no. 4: 609–617. 15282995

[brb371629-bib-0026] Neculicioiu, V. S. , I. A. Colosi , C. Costache , et al. 2023. “Sleep Deprivation‐Induced Oxidative Stress in Rat Models: A Scoping Systematic Review.” Antioxidants 12, no. 8: 1600. 10.3390/antiox12081600. 37627596 PMC10451248

[brb371629-bib-0027] Pankowska, M. M. , H. Lu , A. G. Wheaton , et al. 2023. “Prevalence and Geographic Patterns of Self‐Reported Short Sleep Duration Among US Adults, 2020.” Preventing Chronic Disease 20: E53. 10.5888/pcd20.220400. 37384831 PMC10317035

[brb371629-bib-0028] Ramos, A. R. , A. G. Wheaton , and D. A. Johnson . 2023. “Sleep Deprivation, Sleep Disorders, and Chronic Disease.” Preventing Chronic Disease 20: E77. 10.5888/pcd20.230197. 37651644 PMC10487788

[brb371629-bib-0029] Schoner, W. , and G. Scheiner‐Bobis . 2007. “Endogenous and Exogenous Cardiac Glycosides: Their Roles in Hypertension, Salt Metabolism, and Cell Growth.” American Journal of Physiology‐Cell Physiology 293, no. 2: C509–C536. 10.1152/ajpcell.00098.2007. 17494630

[brb371629-bib-0030] Shi, S. , M. Zhang , W. Xie , et al. 2023. “Sleep Deprivation Alleviates Depression‐Like Behaviors in Mice via Inhibiting Immune and Inflammatory Pathways and Improving Neuroplasticity.” Journal of Affective Disorders 340: 100–112. 10.1016/j.jad.2023.07.119. 37543111

[brb371629-bib-0031] Teleanu, D. M. , A.‐G. Niculescu , I. I. Lungu , et al. 2022. “An Overview of Oxidative Stress, Neuroinflammation, and Neurodegenerative Diseases.” International Journal of Molecular Sciences 23, no. 11: 5938. 10.3390/ijms23115938. 35682615 PMC9180653

[brb371629-bib-0032] Walker, M. P. , and E. van der Helm . 2009. “Overnight Therapy? The Role of Sleep in Emotional Brain Processing.” Psychological Bulletin 135, no. 5: 731–748. 10.1037/a0016570. 19702380 PMC2890316

[brb371629-bib-0033] Wang, C. , Y. Meng , Y. Wang , et al. 2018. “Ouabain Protects Mice Against Lipopolysaccharide‐Induced Acute Lung Injury.” Medical Science Monitor 24: 4455–4464. 10.12659/MSM.908627. 29953424 PMC6053945

[brb371629-bib-0034] Wang, D. , J. Liu , Q. Zhu , et al. 2024. “Ouabain Ameliorates Alzheimer's Disease‐Associated Neuropathology and Cognitive Impairment in FAD4T Mice.” Nutrients 16, no. 20: 3558. 10.3390/nu16203558. 39458551 PMC11510559

[brb371629-bib-0035] Wang, W. , Z. Wang , J. Cao , Y. Dong , and Y. Chen . 2024. “Melatonin Ameliorates Chronic Sleep Deprivation Against Memory Encoding Vulnerability: Involvement of Synapse Regulation via the Mitochondrial‐Dependent Redox Homeostasis‐Induced Autophagy Inhibition.” Free Radical Biology and Medicine 225: 398–414. 10.1016/j.freeradbiomed.2024.10.279. 39396581

[brb371629-bib-0036] Wang, X. , Z. Wang , J. Cao , Y. Dong , and Y. Chen . 2021. “Melatonin Ameliorates Anxiety‐Like Behaviors Induced by Sleep Deprivation in Mice: Role of Oxidative Stress, Neuroinflammation, Autophagy and Apoptosis.” Brain Research Bulletin 174: 161–172. 10.1016/j.brainresbull.2021.06.010. 34144202

[brb371629-bib-0037] Young, J. W. , B. L. Henry , and M. A. Geyer . 2011. “Predictive Animal Models of Mania: Hits, Misses and Future Directions.” British Journal of Pharmacology 164, no. 4: 1263–1284. 10.1111/j.1476-5381.2011.01318.x. 21410454 PMC3229761

[brb371629-bib-0038] Zhang, X. , C.‐W. Liu , X. Sheng , et al. 2025. “Sleep Deprivation Affects Memory Function, Depression and Anxiety‐Like Behaviours in Rats and Mice: A Systematic Review and Meta‐Analysis.” Brain Communications 7, no. 5: fcaf309. 10.1093/braincomms/fcaf309. 40994825 PMC12455040

[brb371629-bib-0039] Zhu, H. , Y. Li , Y. Li , and W. Wang . 2026. “Melatonin Alleviates Retrieval‐Induced Forgetting Deficits in Acute Sleep Deprivation Mice.” Behavioural Brain Research 513: 116315. 10.1016/j.bbr.2026.116315. 42250732

